# Nirvanin

**Published:** 1900-06-15

**Authors:** 


### NIRVANIN.

A French pharmacologist named Jouanin regards Nirvanin as the least toxic of all bodies now employed for local anesthesia, since the lethal dose amounts to ten and one-half grains to two and one-fifth pounds. Reynier confirms this statement of Jouanin, but points out at the same time that the anesthetizing power of nirvanin is visibly less than that of cocain. This shortcoming might, however, be remedied by doubling or even trebling the doses. Extensive experiments have been made with nirvanin in dental surgery. It was employed in the form of subgingival injections as a means of inducing local anesthesia previous to the extraction of teeth by Dumont, Legrand, Stubenrauch, Wittkowoki, Rotenberger, Frick,

Schroeder, Carras and others. The three first named writers express a favorable opinion on the properties of nirvanin, whereas Frick, Schroeder and Carras condemn it, since its injection invariably gives rise in a more or less pronounced degree to pains in the puncture canals and their surroundings, and produce œdematic swellings which are liable to subsist for days.

Regionary anesthesia, according to Oberst, is suitably effected by means of 2 percent solution. One-tenth to five tenths percent solutions should be used in Schleich's method of infiltration. In dental operations it has been usual to inject 2 to 5 percent solutions, using twenty to thirty drops of such solutions. Four to five minutes should be allowed to pass after the injection is made before the extracting is done.—Extract from *Merck's Annual Report.*

